# Salivary proteins offer insights into keratinocyte death during aphthous stomatitis. A case-crossover study

**DOI:** 10.1186/s12903-023-02955-7

**Published:** 2023-05-11

**Authors:** Camila Cofré-Leiva, Paola Andrea Camargo-Ayala, Angela Vergara-Pérez, Romina Hernández-Olivos, Sergio Sanhueza, Estefanía Nova-Lamperti, Jessica Zúñiga-Hernández, César Rivera

**Affiliations:** 1 Departamento de Ciencias Básicas Biomédicas, Facultad de Ciencias de la Salud, Universidad de Talca, Talca, Chile; 2 Laboratorio de Histopatología Oral y Maxilofacial, Departamento de Estomatología, Facultad de Odontología, Universidad de Talca, Avenida Lircay S/N, Campus Norte Universidad de Talca, Talca, Oficina, N°4 Chile; 3 Laboratorio de Inmunología Translacional, Departamento de Bioquímica Clínica e Inmunología, Facultad de Farmacia, Universidad de Concepción, Concepción, Chile

**Keywords:** Recurrent aphthous stomatitis, Proteomics, Endoplasmic reticulum stress, Regulated cell death

## Abstract

**Background:**

The death of oral keratinocytes is a crucial step in the emergence of recurrent aphthous stomatitis (RAS, also known as aphthae or aphthous ulcers). Since there are no experimental models available to research aphthous ulcers, little is understood about this process. We hypothesize that saliva can be a data bank of information that offers insights on epithelial damage.

**Methods:**

In this case-crossover study, we assessed the salivary proteome of patients with RAS (n = 36) in the presence and absence of ulcers using discovery proteomics and bioinformatics. Additionally, we contrasted these patterns with those of healthy individuals (n = 31) who had no prior aphthous ulceration.

**Results:**

Salivary proteome showed that during the ulcerative phase, controlled cell death was downregulated. Due to its ability to distinguish between individuals with and without ulcers, the ATF6B protein raises the possibility that endoplasmic reticulum (ER) stress is responsible for the damage that leads to the death of oral keratinocytes. The high abundance of TRAP1 and ERN1 matches with this biological discovery. The type of death is immunogenic, according to the functional data found in a cell death database.

**Conclusion:**

We identified a cellular process that can lead to the death of oral keratinocytes in the etiopathogenesis process of RAS. Future studies should be conducted to identify what is responsible for the increase in ER stress signaling that would lead to an anti-cell death response.

**Supplementary Information:**

The online version contains supplementary material available at 10.1186/s12903-023-02955-7.

## Background

The most typical ulcerative condition affecting the oral mucosa is recurrent aphthous stomatitis (RAS, also known as aphthae or canker sores). Its defining features are multiple small, recurrent round or ovoid ulcers with circumscribed borders, erythematous haloes, and yellow or gray floors [1]. RAS may be related to systemic conditions such as hypertension [2] and haematinic deficiencies [2, 3], however a causal link has not been confirmed.

Most studies related to RAS are clinical and focus on the disease’s therapy rather than its pathogenesis. The discovery of effective therapeutics has been impeded by a lack of understanding of the specific etiology and mechanism of ulcers. As a result, unlike other common oral diseases, such as periodontal disease, dental caries or oral cancer, RAS cannot be prevented [4].

For the development of the RAS lesion, it is obvious that the oral keratinocyte has to die. Microscopically, in the pre-ulcerative stage, it is possible to observe an infiltration of mononuclear cells that is accompanied by vasculitis and oral keratinocyte vacuolization (an unspecified sign of disease) [5]. Since cell death is a decisive event for the clinical presence of RAS ulcers, very little is known about the process of oral epithelial cell destruction.

Two reports have claimed a role for apoptotic cell death in RAS. The first of them, through electron microscopic examination of oral mucosa of RAS patients, established that the cell morphology was apoptotic [6]. Today it is known that morphology only serves as a very cursory indicator of the sort of cell death [7]. More recently, available data from cell culture and immunohistochemistry suggests that RAS may initiate with abnormal apoptosis of oral epithelial cells, which may progress to secondary necrosis and passive release of damage-associated molecular patterns [8]. These functional inferences were obtained by determining whether molecules like caspase-3 and high mobility group protein B1 (HMGB1) were present or not. Despite this evidence, the cell death pathways involved in RAS are not known and have not been proposed until now.

Our team is interested in deepening the molecular and cellular mechanisms involved in the death of oral keratinocytes. One of the challenges of studying RAS is the lack of a validated experimental model [9]. As RAS ulcers are only histopathologically examined when the diagnosis is unclear, saliva appears in RAS as an alternative to tissue biopsy in the study of this disease directly from patients. Saliva can be utilized as a proxy for the identification of circulating biomarkers for disease and health [10]. Understanding the nature of signaling within the milieu of tissues is possible through analysis of secreted proteome [11]. Since pathological conditions can influence the composition of the salivary proteome [12], we believe that saliva may be a final receptacle for proteins coming from the cells involved in the development of RAS. The salivary proteome has been useful in revealing oral keratinocyte responses in oral cancer [13], Sjögren’s syndrome [14] and oral lichen planus [15]. With these considerations, the objective of this study was to analyze the salivary proteome of RAS patients and assess its usefulness in identifying biological and cellular processes related to the death of oral keratinocytes.

## Methods

### General design

Our study is a case-crossover investigation. Using a timsTOF Pro mass spectrometer coupled to nanoflow liquid chromatography (nLCMS/MS) and computational biology tools, we assessed the salivary proteome of patients with RAS in the presence and absence of ulcers as well as healthy controls. All was carried out in accordance with the Helsinki declaration. This study was authorized by the University of Antofagasta’s Ethics Committee (protocol #156/2018) and Maule Health Service Research Ethics Committee (reference #22-11-2018) 10.6084/m9.figshare.20288775.

### Participants and sample size calculation

We employ a non-probabilistic sampling to incorporate subjects (convenience sample). Volunteers were selected from a cohort of patients with RAS from Centro de Clínicas Odontológicas-Universidad de Talca (Chile). Patients must not have had more than three days’ worth of ulcers at the time of the evaluation. The use of topical or systemic corticosteroids within the month prior to enrolling in the trial, as well as the use of ulcer-treating medications within the previous two days, served as the exclusion criteria. Various exclusion factors included being pregnant, having other types of oral mucosal lesions, diseases with acute or chronic pain, smoking, drinking excessively (more than three times per week), and disorders connected to hematologic deficiencies. Patients with gastrointestinal disorders, immune system disorders, autoinflammatory syndromes, and hematinic deficiencies were also excluded. According to recent recommendations for using mass spectrometry-based proteomics in clinical trials, the number of patients was estimated [16, 17]. We consider a two-sided hypothesis, a power of 80% (1-β = 0.80), and a confidence level of 95% (α = 0.05). Based on log2 differences, changes in protein abundance or effect size (relative intensity data) were taken into consideration (0.5). A magnitude of 0.5 was determined for biological variation, which includes technical variance. Each analysis was done once (proteomics). A minimum of 31 volunteers were required for each disease stage after the participant count was adjusted to account for a potential loss of 20%.

In this case-crossover design, the study population consists of subjects who have experienced RAS (n = 36), who were evaluated during the presence (ulcerative stage) and absence of lesions (remission stage). The study also included healthy controls (n = 31; individuals without a history of RAS ulcers). Furthermore, we collected samples from 15 post-remission recurrences (n = 15), which were not examined by proteomic studies. All RAS ulcers were small (Mikulicz) and developed on non-keratinized mucosa (mostly on the lips). Patients’ data is available at 10.6084/m9.figshare.20288769.

### Saliva collection

Before saliva was collected, subjects were instructed to fast for at least 30 min. This time has proven to be useful when using saliva for diagnostic purposes and for the study of biomarkers [18, 19]. All patients rinsed their mouths with 10 mL of water for 30 s. The collection of whole unstimulated saliva started after 10 min. Between the hours of 8:00 and 11:00 in the morning, each subject continually expectorated into a 50 mL sterile propylene tube for 5 min. Samples were treated with a protease inhibitor mixture and put on ice right away (cOmplete Tablets EASYpack, Roche). The samples were then centrifuged at 14,000xg for 20 min at 4 °C to get rid of cellular debris and undissolved components. The total protein concentration in the supernatants was measured using the BCA Protein Assay (Thermo Scientific). The samples were maintained at -80 °C for later processing. When several persons are being studied, resource limitations can be overcome by pooling samples in proteomics studies [20]. Saliva samples from healthy individuals (*n* = 3, 11 samples per set), people with ulcers (ulcerative stage, *n* = 3, 12 samples per set), and people in the remission phase (*n* = 3, 12 samples per set) were randomly selected and pooled into 9 sets. The BCA Protein Assay kit was used to assess the total protein concentration (Thermo Scientific). Each pooled sample included 70 µg of protein in it.

### Salivary proteome identification and description

We identified salivary proteins using mass spectrometry-based proteomics. Examining 500 ng of peptides following tryptic digestion and peptide separation mass spectrometry data were obtained using a nanoElute LC system coupled to a timsTOF Pro mass spectrometer (Bruker Daltonics). In accordance with previous procedures [21], all MS/MS samples were examined using PEAKS Studio X+ (v.10.5, Bioinformatics Solutions). A protein sequence database of reviewed human proteins with 74,823 entries from UniProt was utilized for all searches, assuming the presence of the digestive enzyme trypsin. The InteractiVenn tool [22] was used to create a Venn diagram that displays the unique and shared proteins from each comparison. The Human Salivary Proteome Wiki (https://www.salivaryproteome.org/), a saliva proteome-focused, open-access database, was used to identify the origin of distinctive proteins [23]. Protein biological function was determined in FunRich software (v. 3.1.4) (accessed 21 June 2020) [24] using hypergeometric analysis where significance level was 0.01. In Funrich we add the list of all identified proteins with the “add dataset” function. In the “gene enrichment” option, we selected “compare” to identify the biological processes that presented the greatest differences between the groups. We chose the top 3 of those comparisons.

### Protein prioritization

Mass spectrometry-based proteomics allows the identification of more than a thousand proteins per sample. To define the most important ones we use computational biology. For protein quantification, we used the spectral counts reported by PEAKS Studio X+. In order to determine the relative quantification of the protein under the various conditions, the number of fragmentation events, or spectral counts, measured for all the peptides belonging to the same protein in each sample were added together [25]. Spectral counts values were normalized through division by column totals and multiplying them by 100 prior to comparative analysis [26]. We prepare a database using the function restructure cases to variables in SPSS statistics (v.23, IBM). In the Perseus software, spectral counts measurements were used to identify differentially abundant proteins between conditions (v.1.6.15.0, Max Planck Institute of Biochemistry) [27]. Minimum valid values in at least one group (3 valid values at 100% in each set) were used to filter the dataset. ANOVA and the Tukey’s HSD post hoc test were used to determine significance (P value < 0.05). Hierarchical clustering analysis of the resulting matrix was performed using a Euclidean distance method. In addition, we perform unstandardized canonical discriminant functions (all and stepwise statistics).

### Proteomics data verification

Because previous studies have established that it is difficult to verify ATF6 family proteins by western blot [28, 29], we established a protocol to achieve their visualization. Saliva samples were randomized and pooled into 9 sets: healthy controls (*n* = 3, 11 samples per set), ulcerative (*n* = 3, 12 samples per set), and remission phase (*n* = 3, 12 samples per set). Briefly, 22 µg of total salivary protein from each set was taken, and it was then put through SDS-PAGE on a 4-20% precast gel (Bio-Rad #456–1093). The anti-ATF6B (1:250, Sigma-Aldrich #HPA046871) primary antibody was incubated overnight for Western and dot blotting. Anti-rabbit HRP-linked antibody (IgG 1:5000, Cell Signaling Technology #7074) was used as secondary antibody. Using the Omega Lum G imaging equipment, proteins were seen using chemiluminescence (Wester Antares, CYANAGEN #XLS142,0250) (Aplegen). Ponceau red was used as a loading control. We chose to use dot blots to directly confirm the presence of protein in each of the samples because the western blot’s results were inconclusive. Using ImageJ (v1.53, National Institutes of Health), we measured the histograms’ darkness to determine the dot blots’ intensity. We utilized ANOVA and the Tukey’s HSD post hoc test after removing outliers. Additionally, we used the Cytokine Bead Array Th1/2/17 Kit (BD) and flow cytometry to assess the levels of interferon gamma (IFN-γ, P01579 Uniprot) and tumor necrosis factor alpha (TNF-α, P01375 Uniprot) in the salivary pools (LSR-Fortessa X-20, BD).

### Relevant proteins involved in cell death and ER stress

We ranked all the protein intensities for each study group, selecting those that were involved in cell death and ER stress activities based on the Uniprot database (https://www.uniprot.org/id-mapping). They were considered relevant if they changed by more than 50 positions on the list. In the ranking, a number nearer 1 denoted higher abundance. This list was taken from the XDeathDB cell death database, which lists 12 different cell death modes at https://pcm2019.shinyapps.io/XDeathDB/.

## Results

### Bottom-up (shotgun) proteomics of saliva reveals a negative regulation of regulated cell death in RAS

**Fig. 1:**
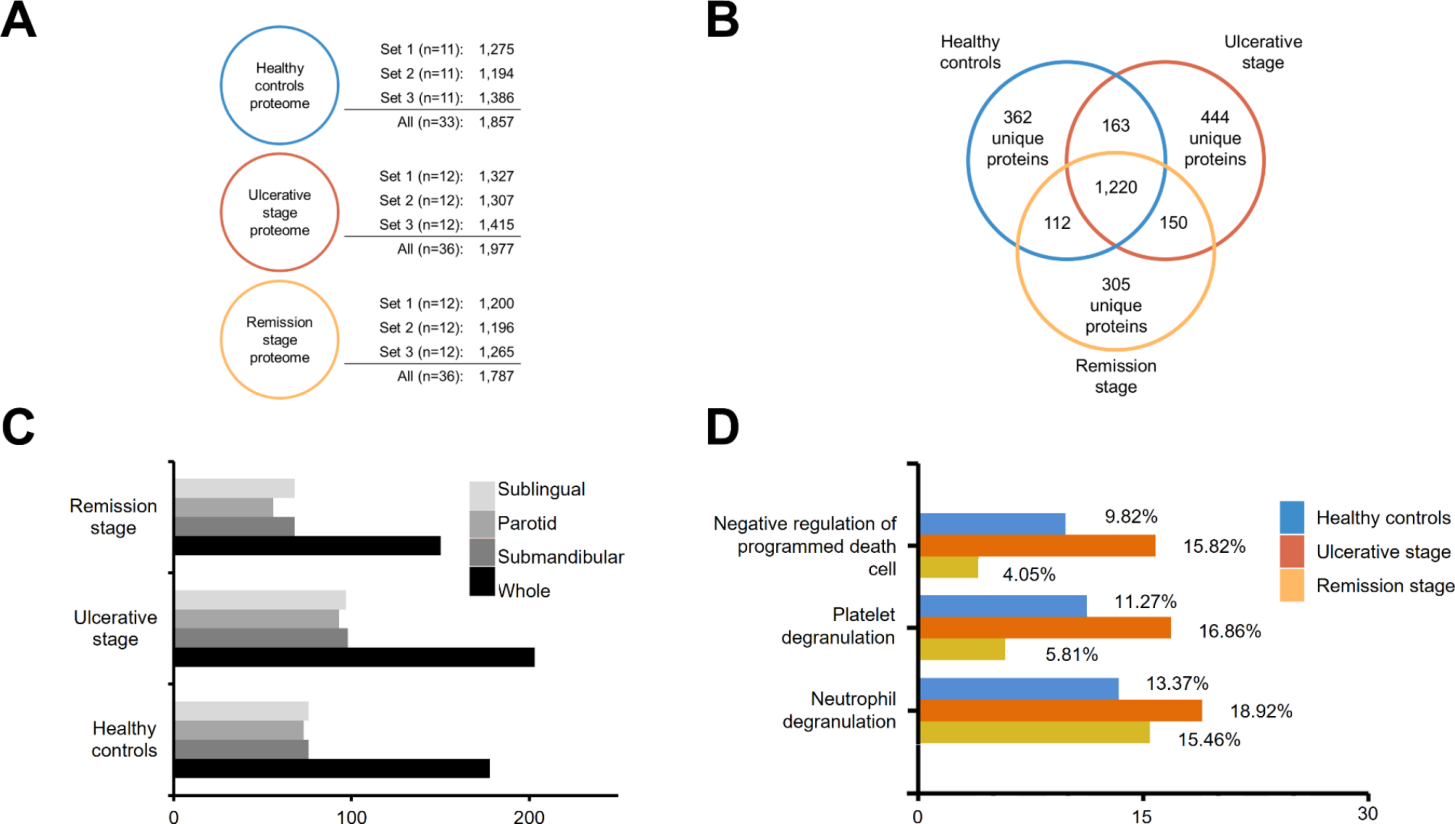
Salivary proteome of recurrent aphthous stomatitis biological processes. (**A**) Circles represent the number of salivary proteins from the respective pooled samples (sets 1, 2 and 3). The numbers on the right indicate the numbers of proteins exactly found in each set. “All” corresponds to all proteins that were detected in at least one set. (**B**) The majority of the salivary proteins discovered were proteins in common, as shown by a Venn diagram. (**C**) Bar graph shows the fraction of saliva to which proteins of each group belong (The Human Salivary Proteome Wiki). (**D**) FunRich categorized the biological processes associated with salivary proteins. Cumulatively, the data show us that there is an increase in the number of proteins involved in the negative regulation of apoptosis

 We believe that saliva may be a repository of proteins that may reflect the biological processes of keratinocytes during RAS. Saliva samples were divided in healthy controls, ulcerative stage and remission stage pooled sets. By a large-scale study of salivary proteins, we identified more than 1,700 proteins per group (Fig. 1A), where the majority were common proteins (Fig. 1B) from all salivary glands (Fig. 1C). To better understand the context in which the protein collective might be participating, we ran a functional analysis. We detected a greater participation of negative regulation of programmed cell death in RAS ulcers than in ulcer-free states (healthy controls and remission stage, Fig. 1D). These results may indicate that the progression of aphthous ulcers may depend, at least in part, on the ability of the oral mucosal epithelium to regulate cell death.

### Differentially abundant proteins distinguish between disease states and those with the best classification profile participate in cell death responses

**Fig. 2:**
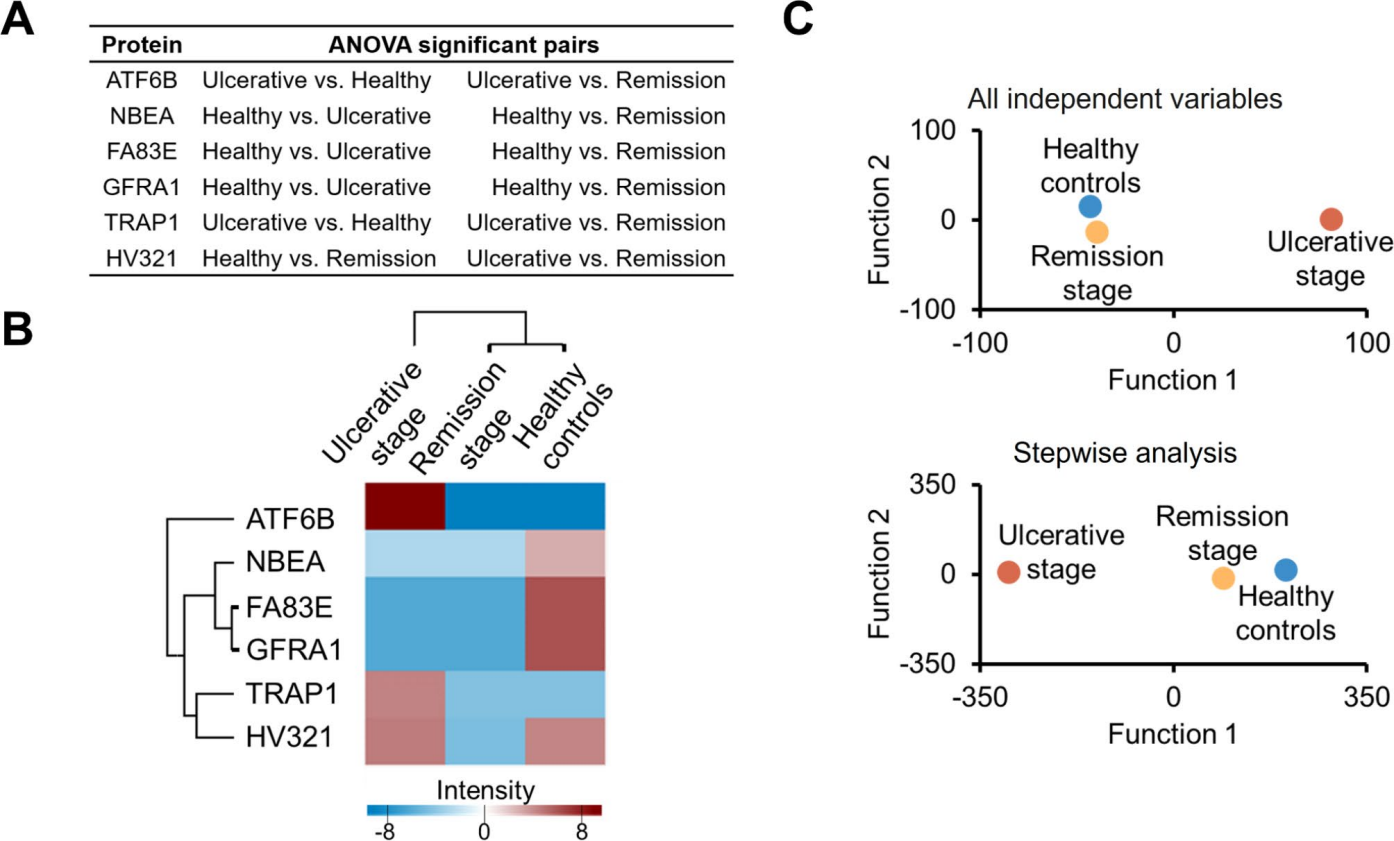
ATF6B provides the best discrimination between the groups. **(A)** Multiple-sample comparison. The table shows the significant comparisons obtained after 1,011 contrasts. In the presence and absence of ulcers, the abundance of ATF6B and TRAP1 are statistically different (ulcerative stage vs. healthy controls, ulcerative stage vs. remission stage). **(B)** Grouping was generated using the Euclidean distance method. Six proteins grouped together the states of absence of aphthous ulcers. ATF6B stands out since it generates a dendrogram on its own. **(C)** The predictive power of proteins was tested with a discriminant analysis. In the upper panel, canonical discriminant functions simultaneously enter all independent variables that satisfy tolerance criteria. All original grouped cases were correctly classified (best ATF6B + and TRAP1-, Wilks’ Lambda < 0.05). FA83E and GFRA1 were not used in the analysis. Bottom panel uses stepwise analysis to control variable entry and removal. All original grouped cases were correctly classified (best TRAP1 + and ATF6B-, Wilks’ Lambda < 0.05). HV321 and GFRA1 were not used in the analysis

Up- and down-regulated proteins have been found to be helpful in bringing proteomics to the clinic [30]. To find out which proteins are differentially abundant between desired states, we compared their expression levels. Protein identification datasets were pre-processed to obtain valid values. This processing resulted in the identification of 1,011 proteins that had 3 valid intensity values in at least one group. The filtered dataset containing 1,011 proteins was subjected to multiple-sample comparison, resulting in 6 proteins showing differential abundance: cyclic AMP-dependent transcription factor ATF6 beta (ATF6B), neurobeachin (NBEA), protein FAM83E (FAM83E), GDNF family receptor alpha-1 (GFRA1), heat shock protein 75 kDa mitochondrial (TRAP1) and immunoglobulin heavy variable 3–21 (HV321) (Fig. 2A). ATF6B and TRAP1 are significantly abundant during the presence of ulcers when compared to the lesion-free groups.

In addition, the proteins permitted the classification of disease states. Our hierarchical clustering analysis shows that protein expression profiles put together healthy controls with remission stage (Fig. 2B). The usage of all 6 proteins together, in theory, might differentiate individual patients. Looking at the dendrograms, ATF6B stands out as the protein with a better classification profile. The difference between ulcerative stage and lesion-free groups was further confirmed with a discriminant function analysis (predictive model for group membership, Fig. 2C). This last analysis showed that TRAP1 is another protein that has a strong potential for group identification.

The 6 proteins identified as the best for group classification are listed in Table 1 with their molecular functions. It is interesting to note that ATF6B and TRAP1 (both proteins with a high abundance in RAS) are involved in endoplasmic reticulum unfolded protein response (UPR). Endoplasmic reticulum (ER) homeostasis disruption causes a stress situation known as “ER stress”, which triggers the UPR, a tightly controlled program whose main goal is to reestablish this organelle’s physiological activity [31]. Activation of UPR is primarily an adaptive response against toxic insults like reactive oxygen species, whereas sustained activation of UPR signaling causes cell death [32].

To explore whether oral keratinocyte death is apoptotic, we examined the levels of salivary TNF-α and IFN-γ using a cytometry bead array. Salivary pools did not contain quantifiable TNF-α and IFN-γ levels (standard curve range was from 19.53 pg/mL to 5,000.00 pg/mL, Supplementary Dataset File 1). It is scientifically conceivable to consider the idea that a flow of these cytokines is not what causes the responses associated with apoptosis and that the type of regulated cell death could be another type.

Collectively these results may indicate that, as a harmful environment increases, oral keratinocytes increase the function of proteins that seek to regulate cellular stress responses and prevent cell death. Destruction of oral epithelial cells, probably through a type of regulated but non-apoptotic death, could make these proteins identifiable in saliva.

**Table 1:** Differentially regulated salivary proteins in RAS.

	Expression			
Protein*	Ulcerative stage	Remission stage	Healthy controls	Molecular function**
ATF6B (Q99941)	(+)	(-)	(-)	Is a transcription factor in the unfolded protein response (UPR) pathway during ER stress.
NBEA (Q8NFP9)	(-)	(-)	(+)	Binds to type II regulatory subunits of protein kinase A and anchors/targets them to the membrane.
FAM83E (Q2M2I3)	(-)	(-)	(+)	Enables protein kinase binding activity. Predicted to be involved in signal transduction. May play a role in MAPK signaling.
GFRA1 (P56159)	(-)	(-)	(+)	Play key roles in the control of neuron survival and differentiation.
TRAP1 (Q12931)	(+)	(-)	(-)	This protein may function in regulating cellular stress responses.
IGHV3-21 (A0A0B4J1V1)	(+)	(-)	(+)	Predicted to be involved in several processes, including activation of immune response; defense response to other organism; and phagocytosis.

*Gene name with Uniprot accession. (+) up-regulated/over-expressed, (−) down-regulated/down-expressed. **We obtained molecular functions from The GeneCards human gene database (https://www.genecards.org/)

### ATF6B is elevated in RAS ulcerative states

**Fig. 3:**
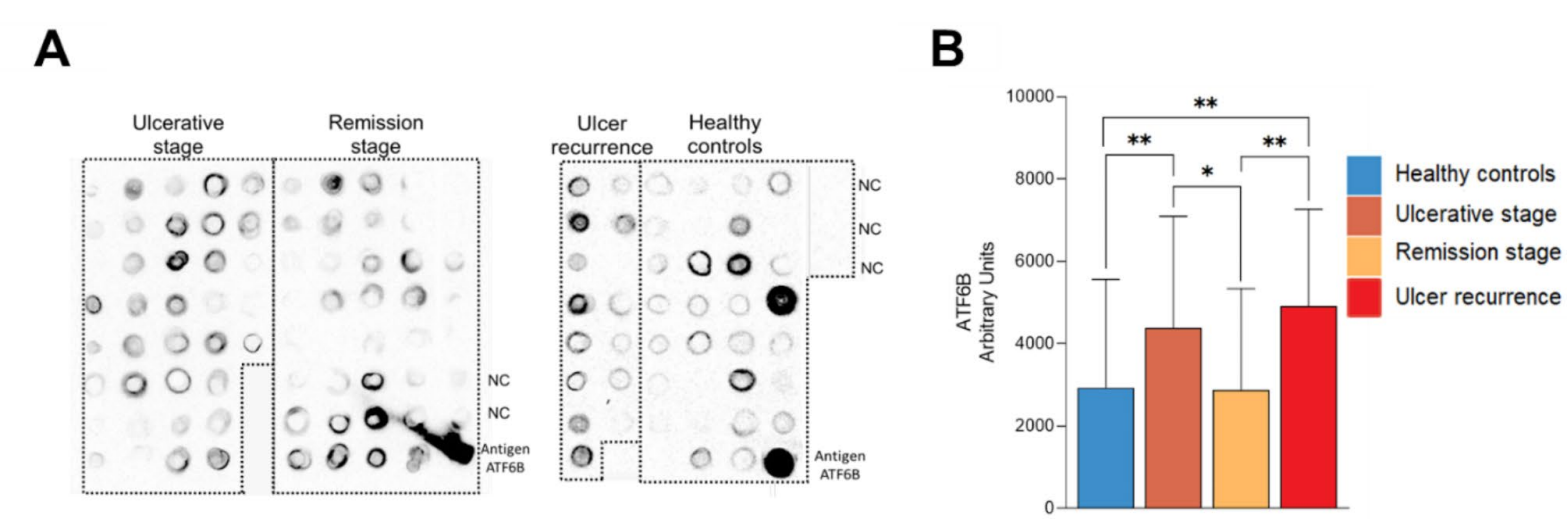
ATF6B is over-expressed in the saliva of RAS patients. During the clinical course of recurrent aphthous stomatitis, salivary ATF6B was confirmed. The ulcerative phase (n = 36) and the remission (healing, n = 36) phase are depicted in the left panels. The recurrence of new ulcers (n = 15) and the healthy controls (n = 31) are shown in the right panels. **(A)** Dot blotting (10 µL/dot) showed that ATF6B was more frequent in ulcerative stage samples (13/15 = 0.86) and less frequently in healthy controls (24/31 = 0.77). As a positive control we used the ATF6b antigen (APREst 83,224, Sigma). NC, negative control. **(B)** Dot blot quantification. Recurrent aphthous stomatitis groups demonstrate more ATF6B staining compared to the control. The multiple comparison between groups showed significant differences *p<0.05, ** p<0.01. The uncropped scans of the gels are shown in Supplementary Fig. 1, dot blot quantification on Supplementary dataset file 2 and western blot analysis result is shown in Supplementary Fig. 2

Because our proteomics information revealed a negative regulation of programmed cell death and that ATF6B (which participates in these responses) is one of the proteins that best contributes to the classification of patient groups, we performed dot blot assays. To further investigate the clinical and biological significance of ATF6B, we examined the association between disease status and protein abundance in all 118 individual samples (Fig. 3A). After removing outliers, the results indicated that high expression of ATF6B was associated with the presence of RAS ulcers (Fig. 3B).

### Protein profiles suggest subclinical inflammation and immunogenic cell death in the RAS cycle

**Fig. 4:**
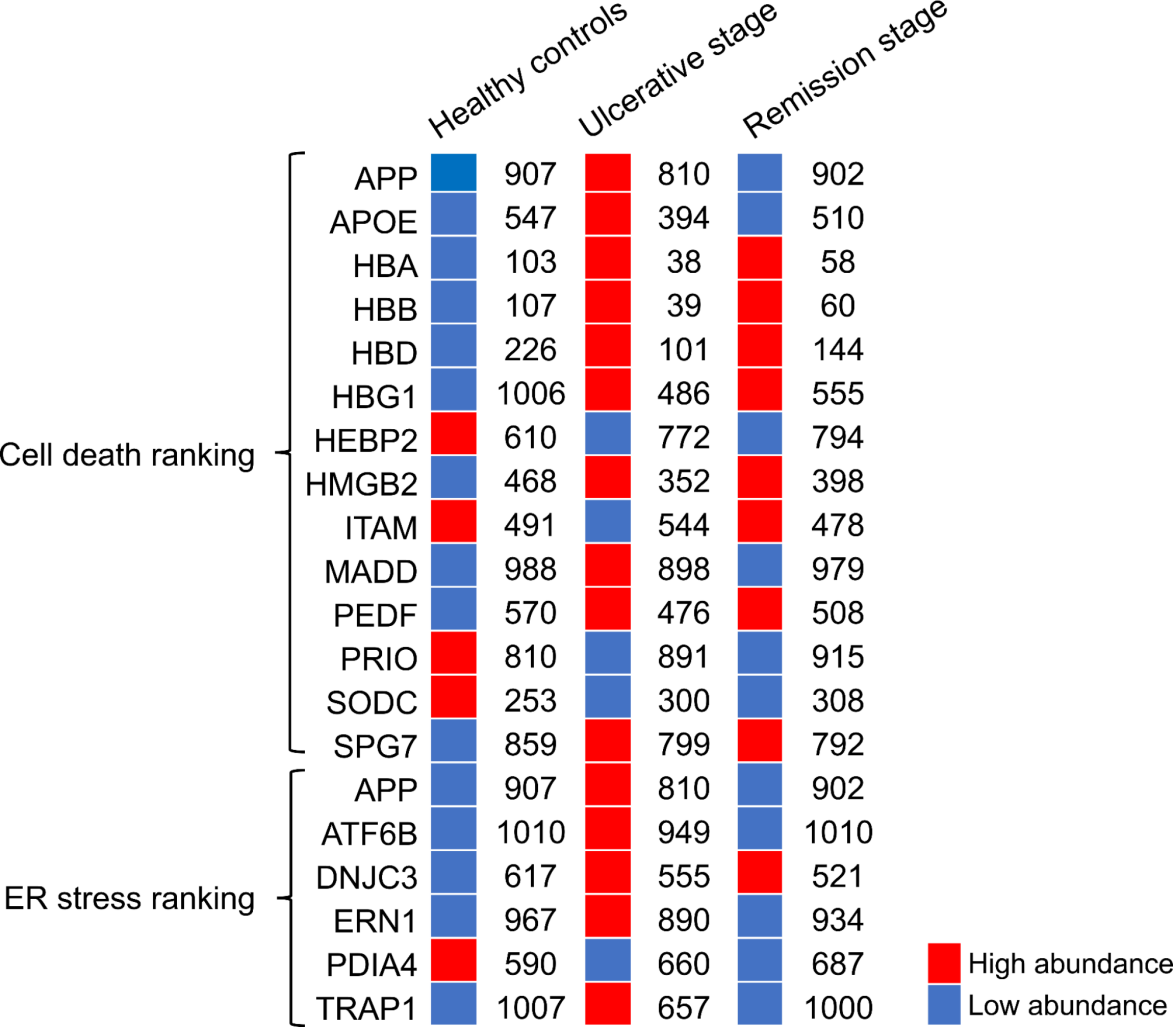
Abundance ranking of proteins involved in cell death and ER stress. The ranking’s possible positions range from 1 to 1,011. ATF6B and ERN1 are involved in parthanatos, a type of programmed cell death, according to the XDeathDB database (https://pcm2019.shinyapps.io/XDeathDB/). Independent of the presence or absence of ulcers, patients with RAS have a similar expression profile. Acute-phase response proteins including HBA, HBB, HBD, and HBG1 continue to be abundant even during remission. This could indicate that subclinical inflammatory phenomena are still present. APP, APOE, MADD, ATF6B, ERN1 and TRAP1 are abundant during the presence of ulcers

We examined the ranking of salivary proteins implicated in cell death and ER stress as prior data collectively demonstrated a response against these processes. To do this we imported the functional annotations of each of the 1,011 proteins from Uniprot. The notable variations between the groups (changes in 50 ranking positions) are shown in Fig. 4. Red squares in the ranking indicated higher abundance; on the other hand, a high number in the ranking indicated lower abundance (blue squares). To more closely understand the link between this ranking and understand the pathogenic mechanisms related to cell death, we consulted the XDeathDB database. Two proteins returned a positive result in the search, ATF6B and serine/threonine-protein kinase/endoribonuclease IRE1 (ERN1). Both proteins participate in a type of cell death called parthanatos, a type of immunogenic cell death.

Most proteins show a similar ranking among people with RAS, independent of the absence or presence of lesions. On this list, proteins hemoglobin subunit alpha (HBA), hemoglobin subunit beta (HBB), hemoglobin subunit delta (HBD) and hemoglobin subunit gamma-1 (HBG1) stand out.

## Discussion

The RAS-specific ulcer is caused by cell loss in the epithelial layer as a result of an etiopathogenic mechanism that is poorly understood. RAS’s effect on the quality of life of those who have the lesions is explained by the magnitude of epithelium damage [33]. In this study, the salivary proteome of RAS patients was analyzed both with and without ulcers, taking into account that the death of the keratinocyte is a crucial event in the development of the disease and that saliva may provide information on the processes linked with this death. The ATF6B protein, which is involved in the negative regulation of cell death by regulating adaptive responses to maintain cell viability, allowed us to distinguish between samples with lesions and those in which the oral mucosa has not been destroyed. Additionally, the differentially abundant proteins suggest that the kind of oral keratinocyte cell death in RAS is immunogenic.

Human saliva will be a useful diagnostic fluid for clinical diagnosis and prognosis soon [34]. We discovered that one of the key biological mechanisms involved in RAS are an increase in platelet and neutrophil degranulation and a negative regulation of programmed cell death, based on more than 1,700 salivary protein identification. The increased platelet activity is to be expected because there is exposure of the connective tissue and underlying vasculature to the epithelium. It has been demonstrated previously that patients with RAS have considerably greater neutrophil counts and neutrophil-to-lymphocyte ratios than healthy controls [35]. A neutrophil-to-lymphocyte ratio has recently been proposed as a reliable mirror of inflammatory status and adaptive immunity [36]. Due to the novelty of inhibition of programmed cell death observed in RAS, we are dedicated to exploring this type of cell death.

The mass spectrometry identification of salivary ATF6B is primarily responsible for the anti-cell death response identified in our investigation. The proteomic results were confirmed by using western blot and dot blot assays, which showed that ATF6B was highly expressed in the presence of RAS ulcers. Protein homeostasis disturbances are caused by a variety of reasons and frequently involve an abnormal accumulation of toxic misfolded proteins, generating a cellular condition known as ER stress. Excess of ER stress activates various cell death pathways [37]. Through processes like the UPR, cells can react to a rapid accumulation of secretory proteins within the ER [38]. During the ER stress response, the ER-transmembrane proteins ATF6-α (ATF6A) and ATF6B are cleaved. The resultant fragments translocate to the nucleus, attach to particular regulatory regions, and affect how ER stress response genes are expressed. This increases cell survival by helping to resolve the stress. Similar to skin regeneration, the expression of ATF6B may indicate that ER stress must be reduced in order for reepithelialization to take place (progress from the ulcerative to the remission stage) [39]. Additionally, TRAP1 (a mitochondrial-specific Hsp90 chaperone) demonstrated a good discrimination potential to discriminate between disease groups. Like ATF6B, this protein is also involved in adaptive responses against cell death. Through controlling protein homeostasis, TRAP1 protects cells from ER stress [40].

Salivary proteomics during RAS suggests that the destruction of oral keratinocytes occurs in an immunogenic-type cell death. In patients with Sjögren’s syndrome, it has been observed that TNF-α and IFN-γ can cause ER stress in the salivary gland acinar cells (an autoimmune disease that destroys exocrine glands). The ATF6A pathway is activated by this induction, preventing apoptosis-related death [29]. TNF-α can initiate the extrinsic pathway of apoptosis by binding to the TNFR1 receptor [41] and may also participate in the intrinsic pathway [42, 43]. Because we were unable to identify quantifiable levels of TNF-α and IFN-γ, we are limited in linking ER stress directly to apoptosis. Regulated cell death may take the form of apoptotic or non-apoptotic. Regulated non-apoptotic cell death is made up of a variety of mechanisms and phenotypes [44]. In order to identify these mechanisms and phenotypes, we ranked the proteins with the most relevant differences in abundance and that participate in cell death processes and ER stress. ATF6B and ERN1 are involved in parthanatos, a kind of cell death, according to the XDeathDB database. Contrary to apoptosis, parthanatos results in large-scale DNA fragmentation rather than the production of apoptotic bodies or small-scale DNA fragmentation. Parthanatos is an immunogenic cell death that involves the loss of cell membrane integrity, similar to necrosis, but unlike it, does not result in cell swelling [45]. Immunogenic cell death refers to a variety of controlled cell death processes that, when stimulated by endogenous antigenic contents from dying or dead cells, result in an improved T cell-dependent immune response [46]. Since immunogenic cell death is highly related to ER stress, frequently leading to an UPR [47], it is plausible to think that this is the type of death that occurs in keratinocytes. This hypothesis should be tested in future functional experiments, because several proteins are shared by different types of regulated cell death.

There is persistence of a proinflammatory signature even in the absence of clinical RAS lesions. Our investigation revealed that the majority of the proteins in the context of cell death and ER-stress share the same expression profile in the ulcerative and remission groups. The persistence of the HBA, HBB, HBD, and HBG1 proteins during full healing of the lesions demonstrates that there is an inflammatory basal state regardless of the disease state. These markers have been presented as circulating proteins involved in the acute-phase response [48] and are differentially expressed when active and resolved molecular phenotypes in pediatric Kawasaki Disease (a form of vasculitis) patients have been compared [48]. The acute-phase response refers to the coordinated series of events that occur nonspecifically in response to local or systemic disturbances (infection, inflammation, or trauma) that work together to reduce tissue damage and speed up the healing process [49]. This, accompanied by the high abundance of amyloid-beta precursor protein (APP), apolipoprotein E (APOE), MADD (MAP kinase-activating death domain protein), ATF6B, ERN1 and TRAP1 in the presence of ulcers, shows that, along with cell death, the epithelial health maintenance response is strong. It is interesting to note that a complete and successful innate immune cell response to an inflammatory or infectious injury has been reported to require APP and/or its cleaved products [50, 51]. MADD has been demonstrated to be essential for MAPK activation and apoptosis prevention following TNF-treatment [52]. The ER transmembrane sensor IRE1 triggers UPR to keep the ER and cells functioning [53]. These findings reinforce the idea of the presence of the epithelial protective response when the ulcer is present.

## Conclusion

Our findings, which combine salivary proteomics, laboratory verification, and computational biology, suggest that ER stress is one of the critical events in the death of oral keratinocytes in the pathogenesis of RAS and that the kind of cell death may be immunogenic. Since the biological inferences were obtained from salivary proteomics, our understanding of the cell death process within the cell mucosa is incomplete. Further research is needed to confirm our findings, for example by incorporating the study of tissue immunohistochemistry, or by performing functional tests that seek to verify and identify the cause of increased ER stress signaling.

Clinically, by understanding the mechanisms of cell death in oral keratinocytes during RAS, potential therapeutic targets for the disease could be identified. For example, drugs that inhibit or modulate specific apoptotic pathways may be effective in treating RAS by reducing the death of oral keratinocytes and promoting tissue healing. Further long-term research with significant independent patient groups and in vitro tests is still necessary to support our findings.

## Electronic supplementary material

Below is the link to the electronic supplementary material.

**Supplementary dataset file 1.** Salivary pools did not contain quantifiable TNF-? and IFN-? levels (standard curve range was from 19.53 pg/mL to 5,000.00 pg/mL)

**Supplementary dataset file 2.** Quantification of each dot blot shown in the 3C figure. Values in bold were removed from the comparison analysis by ANOVA.

**Supplementary Figure 1.** Original blots and gels.

**Supplementary Figure 2.** Western blots.

## Data Availability

This article contains all supplemental materials. The dataset identification for the mass spectrometry proteomic data is PXD026401, and it has been submitted to the ProteomeXchange Consortium through the PRIDE partner repository [54] with the dataset identifier PXD026401 (https://www.ebi.ac.uk/pride/archive/projects/PXD026401). All further information that supports the study’s findings is available upon reasonable request from the corresponding author.

## Abbreviations

*RAS*: recurrent aphthous stomatitis
*ATF6B*: cyclic AMP-dependent transcription factor ATF6 beta
*TRAP1*: heat shock protein 75 kDa mitochondrial
*ERN1*: serine/threonine-protein kinase/endoribonuclease IRE1
*ER*: endoplasmic reticulum
*nLCMS/MS*: timsTOF Pro mass spectrometer coupled to nanoflow liquid chromatography
*IgG*: immunoglobulin G
*IFN-γ*: interferon gamma
*TNF-α*: tumor necrosis factor alpha
*NBEA*: neurobeachin
*FAM83E*: protein FAM83E
*GFRA1*: GDNF family receptor alpha-1
*HV321*: immunoglobulin heavy variable 3–21
*UPR*: unfolded protein response
*TNFR1*: tumor necrosis factor receptor 1
*HBA*: hemoglobin subunit alpha
*HBB*: hemoglobin subunit beta
*HBD*: hemoglobin subunit delta
*HBG1*: hemoglobin subunit delta and hemoglobin subunit gamma-1
*APP*: amyloid-beta precursor protein
*APOE*: apolipoprotein E
*MADD*: MAP kinase-activating death domain protein
*mL*: milliliter
*xg*: times gravity
*°C*: degree Celcius
*µg*: microgram
*ng*: nanogram
*pg*: picogram
