# Supplementary material for: Salivary proteins offer insights into keratinocyte death during aphthous stomatitis. A case-crossover study
Source: BMC Oral Health. 2023 May 11;23:279. doi: 10.1186/s12903-023-02955-7 (PMC10176878; doi:10.1186/s12903-023-02955-7)
Supplement: Supplementary file 1 — Supplementary dataset file 1. Salivary pools did not contain quantifiable TNF-? and IFN-? levels (standard curve range was from 19.53 pg/mL to 5,000.00 pg/mL) [file 12903_2023_2955_MOESM1_ESM.pdf]

Supplementary dataset file 1. Salivary pools did not contain quantifiable TNF-α and IFN-γ levels (standard curve range was from 19.53 pg/mL to 5,000.00 pg/mL

Analyte Name

Bead - Human IFN-γ

Sample Name	Position	Clustering	Sample	Results File	Event #	MFI	SD	CV %	Nominal CC	Fitted CC	Final CC	Unit	Recovery %	Dilution	QC Result	Qualitative	Message
Test002	1 - B2	Auto	Healthy controls	Cesar_C2_P1_002.fcs	129	144.80	66.42	39.60%		0.00	0.00	pg/mL		1.00	N/A	N/A	Fitting: Below
Test003	1 - B3	Auto	Healthy controls	Cesar_C3_P1_003.fcs	107	120.00	37.95	31.75%		0.00	0.00	pg/mL		1.00	N/A	N/A	Fitting: Below
Test004	1 - B4	Auto	Ulcerative stage	Cesar_A1_P1_004.fcs	113	134.40	32.02	25.82%		0.00	0.00	pg/mL		1.00	N/A	N/A	Fitting: Below
Test005	1 - B5	Auto	Ulcerative stage	Cesar_A2_P1_005.fcs	151	113.60	39.14	30.35%		0.00	0.00	pg/mL		1.00	N/A	N/A	Fitting: Below
Test006	1 - B6	Auto	Ulcerative stage	Cesar_A3_P1_006.fcs	121	123.20	35.29	26.58%		0.00	0.00	pg/mL		1.00	N/A	N/A	Fitting: Below
Test007	1 - B7	Auto	Remission stage	Cesar_D1_P1_007.fcs	122	121.60	36.18	28.98%		0.00	0.00	pg/mL		1.00	N/A	N/A	Fitting: Below
Test008	1 - B8	Auto	Remission stage	Cesar_D2_P1_008.fcs	118	112.00	36.32	29.98%		0.00	0.00	pg/mL		1.00	N/A	N/A	Fitting: Below
Test009	1 - B9	Auto	Remission stage	Cesar_D3_P1_009.fcs	116	114.40	32.17	29.53%		0.00	0.00	pg/mL		1.00	N/A	N/A	Fitting: Below
Test010	1 - B10	Auto	Ulcer recurrence	Cesar_R1_P1_010.fcs	143	128.80	41.51	27.78%		0.00	0.00	pg/mL		1.00	N/A	N/A	Fitting: Below
Test011	1 - B11	Auto	Ulcer recurrence	Cesar_R2_P1_011.fcs	119	123.20	32.62	26.26%		0.00	0.00	pg/mL		1.00	N/A	N/A	Fitting: Below
Test012	1 - B12	Auto	Ulcer recurrence	Cesar_R3_P1_012.fcs	121	116.00	41.81	36.06%		0.00	0.00	pg/mL		1.00	N/A	N/A	Fitting: Below
Test021	1 - C9	Manual	Healthy controls	Cesar_C1_001_P1_001.fcs	105	100.00	34.40	29.90%		0.00	0.00	pg/mL		1.00	N/A	N/A	Fitting: Below

Bead - Human TNF

Sample Name	Position	Clustering	Sample	Results File	Event #	MFI	SD	CV %	Nominal CC	Fitted CC	Final CC	Unit	Recovery %	Dilution	QC Result	Qualitative	Message
Test002	1 - B2	Auto	Healthy controls	Cesar_C2_P1_002.fcs	131	204.80	89.55	37.09%		1.76	1.76	pg/mL		1.00	N/A	N/A	Fitting: Below
Test003	1 - B3	Auto	Healthy controls	Cesar_C3_P1_003.fcs	137	116.80	42.11	32.66%		0.00	0.00	pg/mL		1.00	N/A	N/A	Fitting: Below
Test004	1 - B4	Auto	Ulcerative stage	Cesar_A1_P1_004.fcs	131	105.60	46.26	37.02%		0.00	0.00	pg/mL		1.00	N/A	N/A	Fitting: Below
Test005	1 - B5	Auto	Ulcerative stage	Cesar_A2_P1_005.fcs	134	102.40	38.10	31.27%		0.00	0.00	pg/mL		1.00	N/A	N/A	Fitting: Below
Test006	1 - B6	Auto	Ulcerative stage	Cesar_A3_P1_006.fcs	125	92.00	36.18	35.01%		0.00	0.00	pg/mL		1.00	N/A	N/A	Fitting: Below
Test007	1 - B7	Auto	Remission stage	Cesar_D1_P1_007.fcs	140	120.80	35.58	33.68%		0.00	0.00	pg/mL		1.00	N/A	N/A	Fitting: Below
Test008	1 - B8	Auto	Remission stage	Cesar_D2_P1_008.fcs	143	124.00	45.07	35.52%		0.00	0.00	pg/mL		1.00	N/A	N/A	Fitting: Below
Test009	1 - B9	Auto	Remission stage	Cesar_D3_P1_009.fcs	136	104.80	50.85	43.05%		0.00	0.00	pg/mL		1.00	N/A	N/A	Fitting: Below
Test010	1 - B10	Auto	Ulcer recurrence	Cesar_R1_P1_010.fcs	130	102.40	36.62	31.85%		0.00	0.00	pg/mL		1.00	N/A	N/A	Fitting: Below
Test011	1 - B11	Auto	Ulcer recurrence	Cesar_R2_P1_011.fcs	124	92.00	39.44	36.76%		0.00	0.00	pg/mL		1.00	N/A	N/A	Fitting: Below
Test012	1 - B12	Auto	Ulcer recurrence	Cesar_R3_P1_012.fcs	133	112.80	53.97	42.52%		0.00	0.00	pg/mL		1.00	N/A	N/A	Fitting: Below
Test021	1 - C9	Manual	Healthy controls	Cesar_C1_001_P1_001.fcs	85	87.20	32.02	33.45%		0.00	0.00	pg/mL		1.00	N/A	N/A	Fitting: Below
