# Supplementary material for: Salivary proteins offer insights into keratinocyte death during aphthous stomatitis. A case-crossover study
Source: BMC Oral Health. 2023 May 11;23:279. doi: 10.1186/s12903-023-02955-7 (PMC10176878; doi:10.1186/s12903-023-02955-7)
Supplement: Supplementary file 2 — Supplementary dataset file 2. Quantification of each dot blot shown in the 3C figure. Values in bold were removed from the comparison analysis by ANOVA. [file 12903_2023_2955_MOESM2_ESM.pdf]

Supplementary dataset file 2. Quantification of each dot blot shown in the 3C figure. Values in bold were removed from the comparison analysis by ANOVA.

HEALTHY CONTROLS

	1	2	3	4
A	5.363,497	511,577	451,527	2.194,912
B	3.506,205	174,042	4.771,255	155,021
C	6.696,690	9.592,134	**11.849,912**	949,891
D	6.851,518	1.974,598	1.119,355	**18.682,933**
E	6.600,468	3.850,891	1.920,962	803,527
F	3.974,640	238,406	8.903,497	547,698
G	2.225,397	417,255	2.029,912	1.317,305
H	710,255	4.061,548	1.498,598	

REMSSION STAGE

	1	2	3	4	5
A	1.619,477	**9.946,134**	3.111,962	695,184	788,447
B	2.378,891	3.902,154	1.087,527	446,991	784,326
C	421,113	724,648	2.207,376	6.441,083	**9.876,874**
D	247,213	4.009,276	1.843,134	6.974,480	3.025,175
E	490,355	240,950	446,627	581,163	
F	734,062	1.359,598	6.297,962	870,527	
G	5.938,104	6.693,912	**12.009,225**	**8.089,276**	
H	577,790	**11.385,912**	5.704,426	6.832,933	

ULCERATIVE STAGE

	1	2	3	4	5
A	834,941	5.697,397	1.167,548	8.921,154	**13.063,246**
B	1.426,598	2.033,205	7.305,205	7.020,326	**20.464,045**
C	**474,548**	3.421,205	**10.756,083**	6.901,790	2.436,589
D	**9.649,447**	4.375,033	5.613,205	1.109,012	2.634,853
E	2.036,062	5.552,983	3.879,326	7.723,619	11.069,610
F	2.155,255	9.776,640	4.346,326	2.865,033	
G	**376,406**	503,991	1.365,184	2.397,891	
H	1.481,820	**365,870**	4.826,497	7.694,841	

RECURRENCE STAGE

	1	2
A	5.405,376	2.865,518
B	10.937,790	**11.740,054**
C	2.167,820	**270,304**
D	**10.704,669**	4.716,104
E	4.749,719	2.963,983
F	3.538,891	4.938,882

G	4.760,255	2.001,882
H	8.636,790	

NEGATIVE (NC) AND POSITIVE (PC) CONTROLS

25,536	NC
33,243	NC
229.534,560	PC

We used the Image J software to quantify the dot blot assays and the descriptive statistics were performed (GraphPad Prism 8.0.1 software, GraphPad Software, Inc. San Diego, USA), we performed a one-way ANOVA, extreme values were removed from the analysis for graphing (shown in bold in the table) and the Benjamini Krieger an Yekutiel test assessed the statistical significance of differences between mean values.

	Ordinary one-way ANOVA<br>Multiple comparisons						
1	Number of families						
2	Number of comparisons per family						
3	Q						
4							
5	**Two-stage linear step-up procedure of Benjamini, Krieger and Yekutieli**	**q value**	**Individual P Value**				
6	CONTROL vs. ULCERA	0.0124	0.0237	A-B			
7	CONTROL vs. REMISSION	0.3274	0.9356	A-C			
8	CONTROL vs. RECURRENCIA	0.0111	0.0155	A-D			
9	ULCERA vs. REMISSION	0.0111	0.0158	B-C			
10	ULCERA vs. RECURRENCIA	0.2108	0.5020	B-D			
11	REMISSION vs. RECURRENCIA	0.0111	0.0114	C-D			
12							
13	**Test details**	**Mean Diff.**	**SE of diff.**	**n1**	**n2**	**t**	**DF**
14	CONTROL vs. ULCERA	-1455	634.4	31	35	2.293	112
15	CONTROL vs. REMISSION	51.41	634.4	31	35	0.08103	112
16	CONTROL vs. RECURRENCIA	-1990	809.1	31	15	2.459	112
17	ULCERA vs. REMISSION	1506	614.9	35	35	2.450	112
18	ULCERA vs. RECURRENCIA	-534.7	793.9	35	15	0.6736	112
19	REMISSION vs. RECURRENCIA	-2041	793.9	35	15	2.571	112
20							
