# Supplementary material for: Salivary proteins offer insights into keratinocyte death during aphthous stomatitis. A case-crossover study
Source: BMC Oral Health. 2023 May 11;23:279. doi: 10.1186/s12903-023-02955-7 (PMC10176878; doi:10.1186/s12903-023-02955-7)

Supplementary figure. Uncropped scans of gels and blots

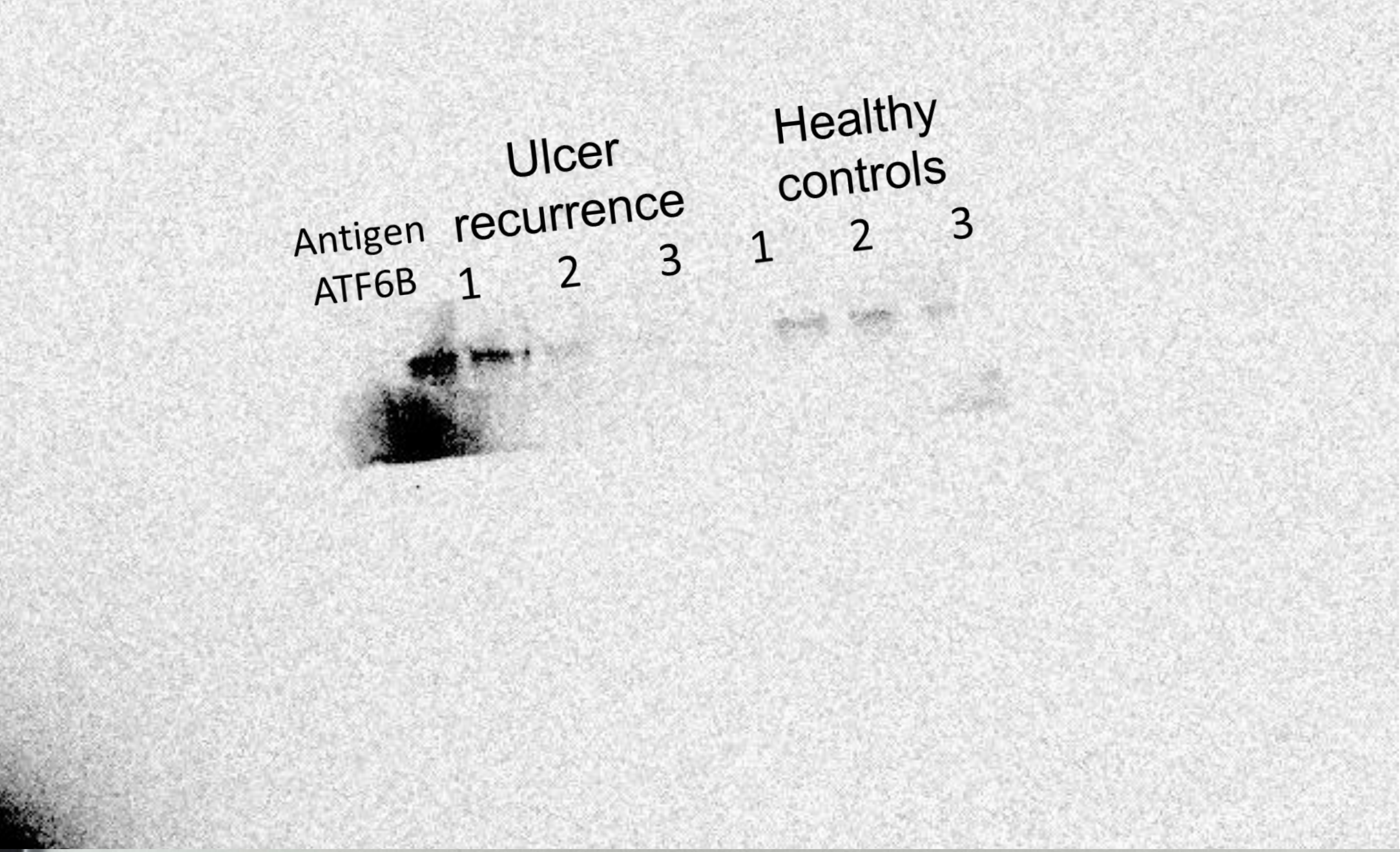

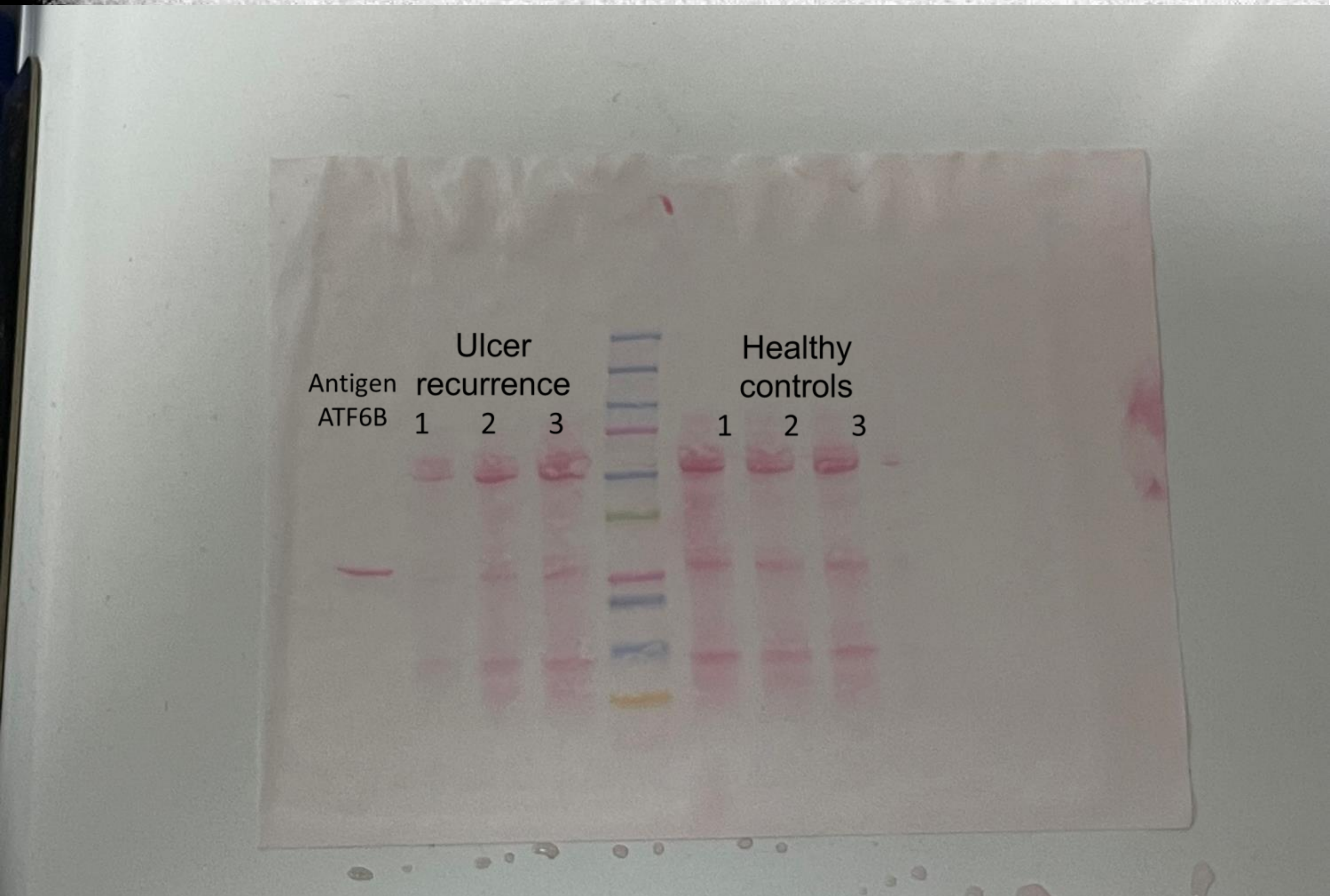

Supplementary figure. Uncropped scans of gels and blots

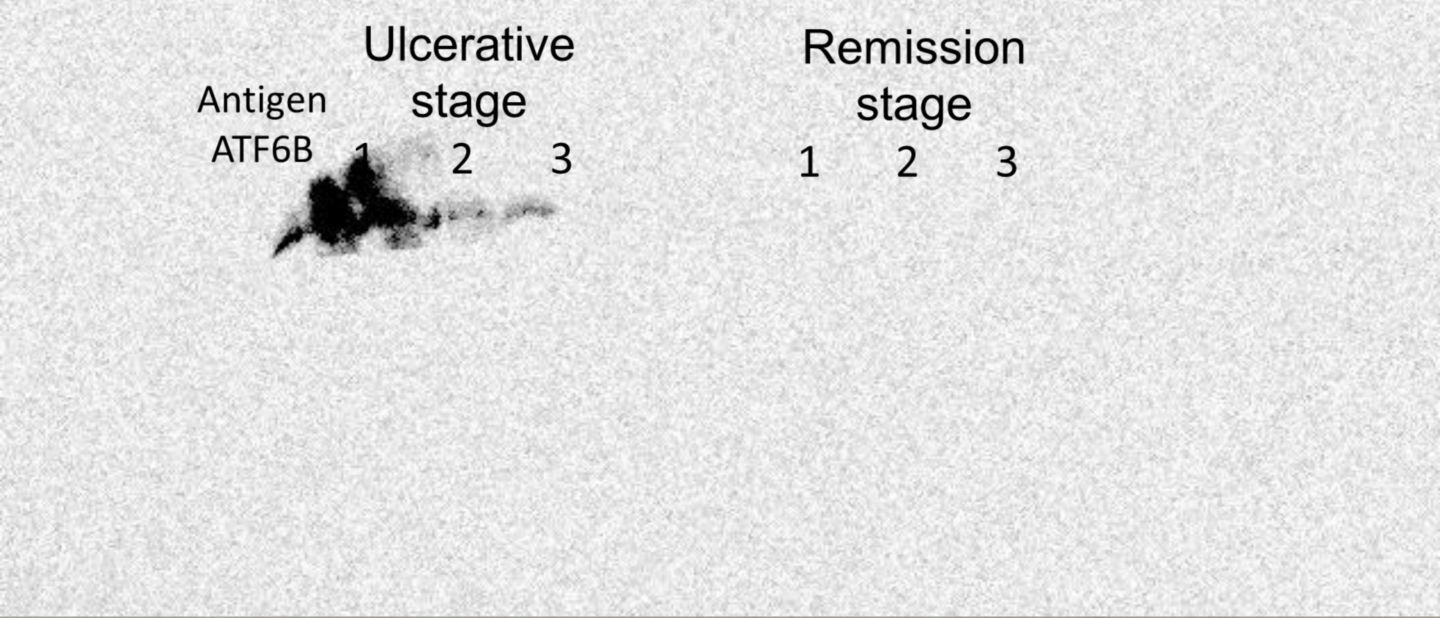

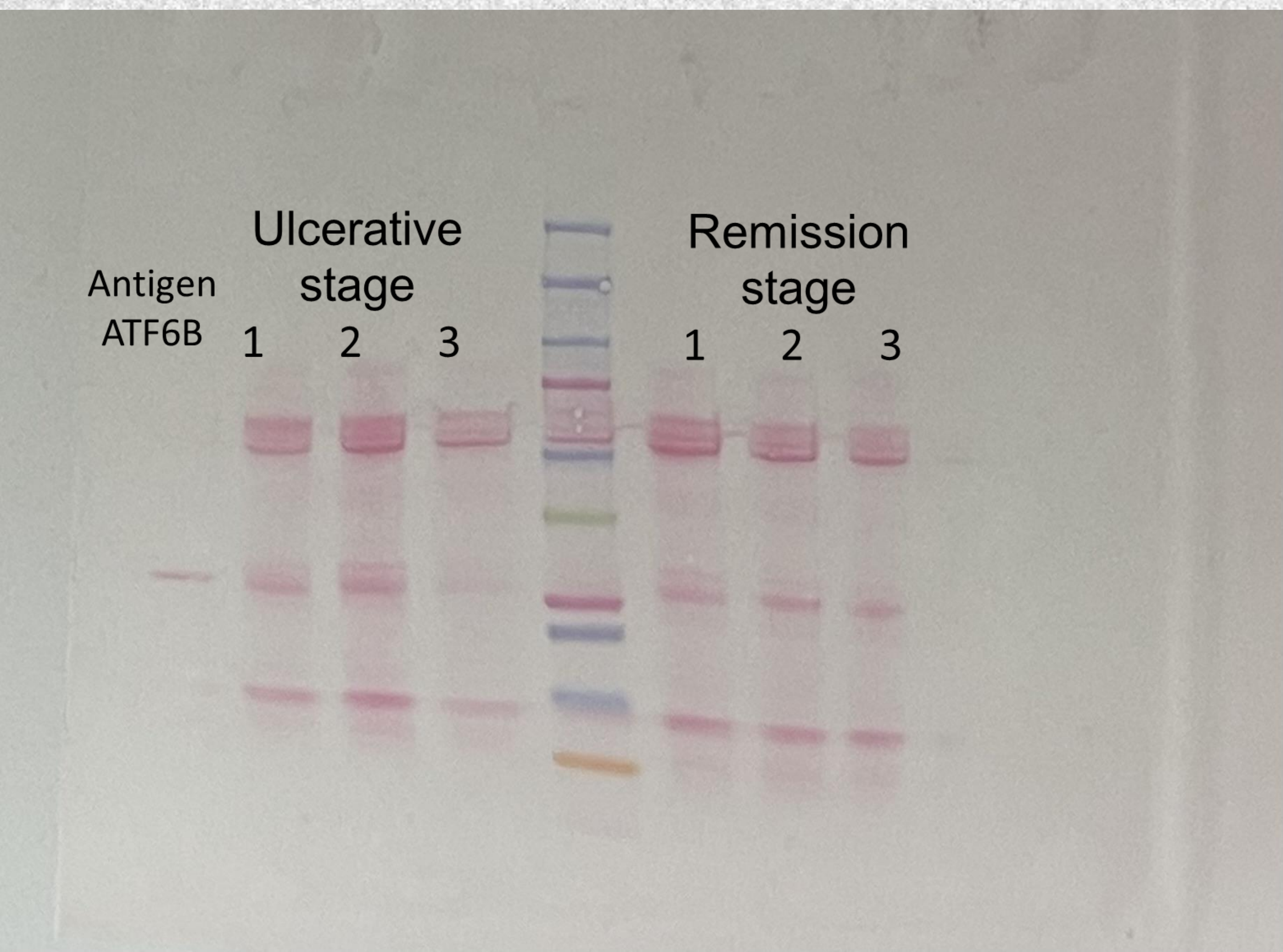

Supplement: Supplementary file 3 — Supplementary Figure 1. Original blots and gels. [file 12903_2023_2955_MOESM3_ESM.pdf]
