# Supplementary material for: Salivary proteins offer insights into keratinocyte death during aphthous stomatitis. A case-crossover study
Source: BMC Oral Health. 2023 May 11;23:279. doi: 10.1186/s12903-023-02955-7 (PMC10176878; doi:10.1186/s12903-023-02955-7)
Supplement: Supplementary file 4 — Supplementary Figure 2. Western blots. [file 12903_2023_2955_MOESM4_ESM.pdf]

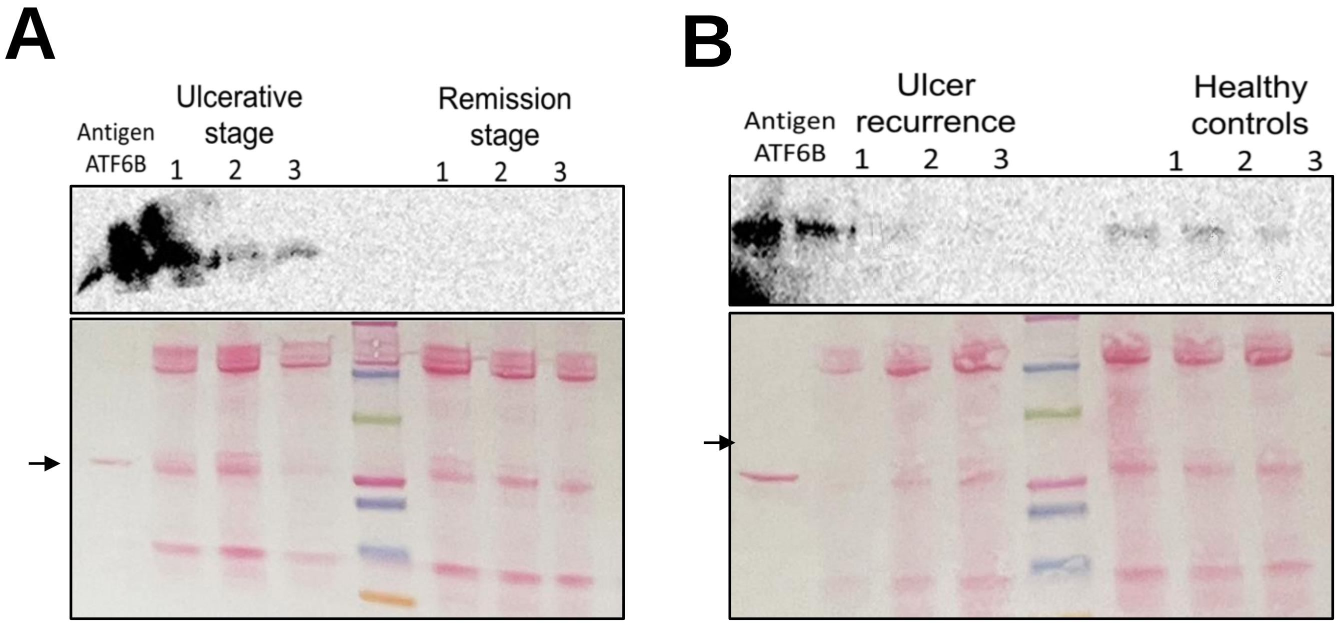

Supplementary figure 2. (A-B) ATF6B protein levels were higher during the ulcerative stages, according to western blotting (pooled samples, 22 μg/lane protein concentration). The loading control used was Ponceau red below panel. The samples to the ulcerative phase and the remission (healing) phase are depicted in the left panels. The recurrence of new ulcers and the healthy controls are shown in the right panels. Previous research has discussed the challenge of obtaining a western blot of ATF6B family proteins [1, 2].

Refs.

1. Yang H, Niemeijer M, van de Water B, Beltman JB. ATF6 Is a Critical Determinant of CHOP Dynamics during the Unfolded Protein Response. iScience. 2020;23:100860.
2. Pro-inflammatory cytokines enhance ERAD and ATF6α pathway activity in salivary glands of Sjögren's syndrome patients. J Autoimmun. 2016;75:68–81.
